# Pulmonary Deposition of Radionucleotide-Labeled Palivizumab: Proof-of-Concept Study

**DOI:** 10.3389/fphar.2020.01291

**Published:** 2020-08-19

**Authors:** Anushi E. Rajapaksa, Lien Anh Ha Do, Darren Suryawijaya Ong, Magdy Sourial, Duncan Veysey, Richard Beare, William Hughes, William Yang, Robert J. Bischof, Amarin McDonnell, Peter Eu, Leslie Y. Yeo, Paul V. Licciardi, Edward K. Mulholland

**Affiliations:** ^1^ New Vaccines, Murdoch Children’s Research Institute, Parkville, VIC, Australia; ^2^ Neonatal Research, Royal Children’s Hospital, Parkville, VIC, Australia; ^3^ Newborn Research, Royal Women’s Hospital, Parkville, VIC, Australia; ^4^ Department of Paediatrics, University of Melbourne, Parkville, VIC, Australia; ^5^ Animal Model Unit, The Royal Children’s Hospital, Parkville, VIC, Australia; ^6^ Nuclear Imaging, The Royal Children’s Hospital, Parkville, VIC, Australia; ^7^ Developmental Imaging, Murdoch Children’s Research Institute, Parkville, VIC, Australia; ^8^ Department of Medicine, Monash University, Melbourne, VIC, Australia; ^9^ The Ritchie Centre, Hudson Institute of Medical Research, Clayton, VIC, Australia; ^10^ School of Health and Life Sciences, Federation University, Berwick, VIC, Australia; ^11^ School of Engineering, Royal Melbourne Institute of Technology, Melbourne, VIC, Australia; ^12^ Department of Cancer Imaging, Peter MacCallum Cancer Centre, Parkville, VIC, Australia; ^13^ Department of Disease Control, London School of Tropical Medicine and Hygiene, London, United Kingdom

**Keywords:** respiratory syncytial virus, lamb model, prophylactic, monoclonal antibody, palivizumab, nebulization

## Abstract

**Objective:**

Current prevention and/or treatment options for respiratory syncytial virus (RSV) infections are limited as no vaccine is available. Prophylaxis with palivizumab is very expensive and requires multiple intramuscular injections over the RSV season. Here we present proof-of-concept data using nebulized palivizumab delivery as a promising new approach for the prevention or treatment of severe RSV infections, documenting both aerosol characteristics and pulmonary deposition patterns in the lungs of lambs.

**Design:**

Prospective animal study.

**Setting:**

Biosecurity Control Level 2-designated large animal research facility at the Murdoch Children’s Research Institute, Melbourne, Australia.

**Subjects:**

Four weaned Border-Leicester/Suffolk lambs at 5 months of age.

**Interventions:**

Four lambs were administered aerosolized palivizumab conjugated to Tc-99m, under gaseous anesthesia, using either the commercially available AeroNeb Go^®^ or the investigational HYDRA device, placed in-line with the inspiratory limb of a breathing circuit. Lambs were scanned in a single-photon emission computed tomography (SPECT/CT) scanner in the supine position during the administration procedure.

**Measurements and Main Results:**

Both the HYDRA and AeroNeb Go^®^ produced palivizumab aerosols in the 1–5 µm range with similar median (geometric standard deviation and range) aerosol droplet diameters for the HYDRA device (1.84 ± 1.40 μm, range = 0.54–5.41μm) and the AeroNeb Go^®^ (3.07 ± 1.56 μm, range = 0.86–10 μm). Aerosolized palivizumab was delivered to the lungs at 88.79–94.13% of the total aerosolized amount for all lambs, with a small proportion localized to either the trachea or stomach. No difference between devices were found. Pulmonary deposition ranged from 6.57 to 9.25% of the total dose of palivizumab loaded in the devices, mostly in the central right lung.

**Conclusions:**

Aerosolized palivizumab deposition patterns were similar in all lambs, suggesting a promising approach in the control of severe RSV lung infections.

## Introduction

Respiratory syncytial virus (RSV) is the leading pathogen causing lower respiratory tract infections (O’Brien et al., 2019), and has been responsible for up to 199,000 deaths worldwide in children under 5 years old annually (Nair et al., 2010). Since 2013, the World Health Organization has designated protection against severe RSV disease as a high priority, particularly for infants <6 months of age, preterm infants, and infants with underlying comorbidities. There is no vaccine available, and palivizumab, a humanized monoclonal antibody (mAb) against RSV F protein, is currently the only licensed preventive product but is costly, limited in effectiveness and requires monthly intramuscular injections.

Inhaled delivery is one approach to provide pain- and needle-free administration of palivizumab. It also offers the advantage of delivering the mAb immediately and directly to the respiratory tract and lung, potentially providing a rapid clinical benefit for the prevention and/or treatment of severe RSV infection. Our recently developed acoustic nebulizer, HYDRA (HYbriD Resonant Acoustics) (Rezk et al., 2016) allows for fast and effective aerosol delivery of mAbs (Cortez-Jugo et al., 2015) and other large biological molecules such as DNA (Rajapaksa et al., 2014) to the respiratory surface *via* inhalation. Using a lamb model, we compare the deposition of aerosolized palivizumab to the lungs using the HYDRA or the commercially-available AeroNeb Go^®^ (now marketed as Innospire Go by Philips Respironics) vibrating mesh nebulizer as a proof-of-concept approach for the prevention and/or treatment of severe RSV disease.

## Materials and Methods

Laser diffraction (Spraytec^®^, Malvern Instruments, Malvern, UK) was employed to determine the aerodynamic diameter (D_ae_) of the aerosols produced using the HYDRA or the AeroNeb Go^®^ (Aerogen, Galway, Ireland), from which the median aerosol size (D_v50_) was calculated from a volume-based size distribution. The geometric standard deviation (GSD) was manually calculated using standard methodology (Finlay, 2019).

The lamb study was approved by the Murdoch Children’s Research Institute (MCRI) Animal Ethics Committee. Experiments were designed and reported with reference to the ARRIVE (Animal Research: Reporting of *in vivo* Experiments) guidelines (Kilkenny et al., 2010). Four month-old lambs were given 60 mg of palivizumab (Synagis^®^, AbbVie, Illinois, USA) at 10 mg/ml, conjugated to a radiotracer technetium-99m (Tc-99m; Global Medical Solutions, Tullamarine, Australia). ^99m^TcO_4_-palivizumab was prepared by a ligand exchange method using glucoheptonate and tin chloride (SnCl_2_). Briefly, 1 ml of palivizumab (100 mg/ml) was diluted in 1 ml water for injection, then incubated at room temperature with 150 μl of 2-mercaptoethanol for 20 min, before purification through a PD-10 size exclusion column (30,000 MWCO). To this purified eluent, 100 μl of glucoheptonate/SnCl_2_ stock (0.2 M glucoheptonate, 0.002 M SnCl_2_) solution was added and mixed. Roughly, 0.5 MBq of ^99m^TcO_4_ was then added to the palivizumab/glucoheptonate solution and incubated for 30 min at room temperature. Purity was checked using the iTLC-SG method in water, before final dilution of the conjugate to 10 ml (10 mg/ml palivizumab).

Palivizumab aerosols were delivered *via* an endotracheal tube (Smiths Medical, Minnesota, USA) to spontaneously breathing lambs (n = 2 lambs for each device) under anesthesia placed in prone position. Pulmonary deposition was mapped using single-photon emission computed tomography and computed tomography (SPECT/CT) using a Symbia Intevo 16 scanner (Siemens, Munich, Germany).

Lambs were placed in the prone position for this procedure. SPECT portions of the scans were performed using the following SPECT parameters: 128 × 128 matrix, 1.23× zoom, Tc-99m NMG camera pre-set, 25 s per view for 64 views using a 180° detector rotation and continuous scan mode. CT portions of the scans were performed following topogram Scout view and imaged at 20mA and 80kV. Image slices of 3 mm thickness were reconstructed using a I50s Medium Sharp kernel with a lung window. Pre- and post-delivery radioactive counts were measured to determine efficiency of the aerosolized palivizumab administration. Briefly, for the SPECT/CT analysis, lung structure segmentation was performed using morphological watersheds with manually placed seeds applied to the gradient of the CT image. Regional distribution of pulmonary deposition was determined by voxelwise summation within each lung structure. The efficiency of palivizumab delivery to lambs relative to the total palivizumab prepared and the efficiency of palivizumab delivery to the lungs relative to the whole body are presented as geometric means (%). A RSV neutralization assay (Do et al., 2019) was used to determine the bioactivity of palivizumab before and after aerosolization.

## Results

**Figure 1:**
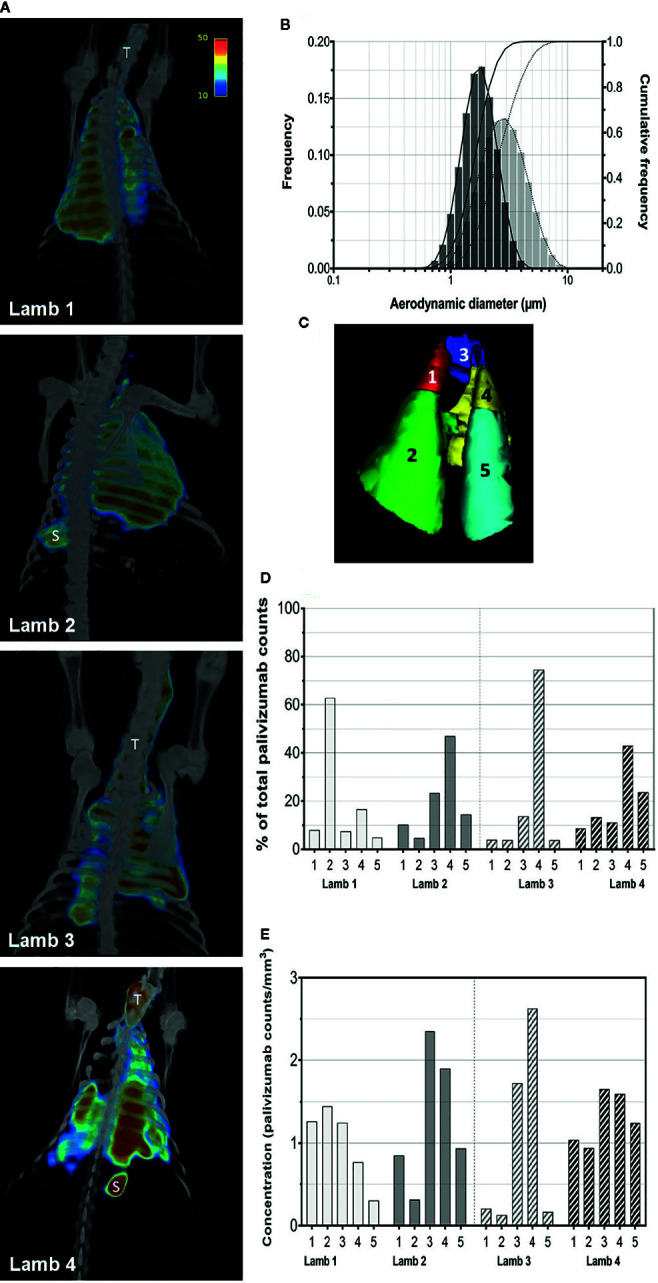
Aerosolization and pulmonary deposition of palivizumab in lambs. **(A)** Reconstructed SPECT/CT images are shown for lambs 1 and 2 administered palivizumab using AeroNeb Go^®^ and lambs 3 and 4 using the HYDRA device. The trachea (T) and stomach (S) were also visible in some deposition images. **(B)** Volume frequencies of the palivizumab aerosol size at given aerodynamic diameters for the AeroNeb Go^®^ (light bars, dotted lines) and HYDRA device (dark bars, solid lines). **(C)** Lungs were segmented into left apical (1), left caudal (2), right apical (3), right middle (4), and right caudal (5) lobes as shown. **(D)** Regional deposition patterns of aerosolized palivizumab in individual lambs relative to the whole lung. **(E)** Concentration of palivizumab aerosols (counts/mm^3^) are shown within individual lobes. Values on the intensity scale correspond to palivizumab density (counts per voxel).

Efficiency of palivizumab conjugation to Tc-99m was >90% (**Table 1**). The median aerosol droplet diameter for palivizumab were similar for both the HYDRA device (1.84 ± 1.40 μm, range = 0.54–5.41 μm) and the AeroNeb Go^®^ (3.07 ± 1.56 µm, range = 0.86–10 μm) nebulizers, producing palivizumab aerosols in the 1–5 µm range that is optimal for deep lung deposition (**Figure 1A**). The RSV neutralizing activity of conjugated palivizumab before and after aerosolization was similar to native palivizumab and comparable between the devices (**Table 2**).

**Table 1:** Conjugation efficiency of palivizumab conjugated to technetium-99m (Tc-99m).

Lamb	Device	% palivizumab deposited in lungs	
		Reading 1	Reading 2
1	AeroNeb Go^®^	98.51	98.33
2	AeroNeb Go^®^	97.94	91.75
3	HYDRA	94.83	90.32
4	HYDRA	99.09	98.41

**Table 2:** Efficiency of aerosolized palivizumab delivery using the AeroNeb Go^®^ and HYDRA devices.

Lamb	Device	NAb ED_50_ titre (Mean ± SD)	% palivizumab delivered to lamb	% palivizumab deposited in lungs
1	AeroNeb Go^®^	11.35 ± 0.1^a^	8.8	88.79
2	AeroNeb Go^®^		9.25	91.86
3	HYDRA	11.39 ± 0.1^a^	ND^b^	94.13
4	HYDRA		6.57	92.51

Data shown for individual lambs.

NAb, neutralizing antibody; ED50, titre of antibody that neutralises 50% of RSV infection; ^a^Mean (± SD) NAb titre of native palivizumab stock (15.45 ± 0.3); ^b^ND, not determined due to failure in taking pre-aerosolization measurements in one portion of the SPECT scanner.

Analyses of pulmonary deposition data from the SPECT/CT images showed the percentage of total aerosolized palivizumab delivered to the lambs (relative to that loaded to the devices) ranged from 6.57 to 9.25% (**Table 2** and **Figure 1B**). A large percentage of the palivizumab aerosols (88.79–94.13%) was deposited in the lungs (**Table 1**) with only a small proportion localized to either the trachea or stomach. A similar level of deposition was found for both devices (**Table 2**). Regional deposition bias was demonstrated towards the left lung for lamb 1 and the right lung for all other lambs, with deposition detected across all five lung segments (**Figures 1C–E**; see also the **Supplementary Videos**).

## Discussion

This is the first study, to our knowledge, to explore the pulmonary deposition of aerosolized palivizumab. We used SPECT/CT as it is superior to planar imaging by providing a three-dimensional assessment of aerosol deposition, allowing determination of intrapulmonary distribution and precise anatomical regional measurements. We were able to accurately visualize palivizumab deposition in the lungs of all four lambs regardless of the aerosolization device employed, providing proof-of-concept for aerosol delivery of a RSV-specific mAb to the potential sites of RSV replication.

Both the AeroNeb Go^®^ and HYDRA devices showed similar aerosol deposition in the lungs relative to the whole body, without detriment to the palivizumab activity. The HYDRA can potentially generate smaller particle size distributions without the requirement of a mesh but we used a slightly reduced nebulization rate compared with Aeroneb Go^®^ for large biomolecules such as palivizumab.

While significant loss of palivizumab due to deposition in the endotracheal tube was observed, we do not expect the tube to have any impact on the differences observed. In three lambs, we noticed a depositional bias towards the right lung, with two of these lambs showing higher deposition efficiency in the right upper lobes in contrast to the other lamb which had higher deposition in the lower left lobe. Several factors may explain the difference observed in lamb 1: (1) maintaining procedural anesthesia while receiving brief periods of manual ventilation and (2) scheduling differences with the BALF procedure (2 weeks earlier due to technical difficulties) could have induced fluid bias to the right lung. A possible explanation might be that the infusion of saline into the lung cavity during BALF collection promoted increased humidity in the respiratory tract, which has been shown to alter aerosol size and movement patterns (Cheng, 2014). As such the humid conditions of the right lung may have attracted more aerosols into the respective lobes.

Targeting the lungs is proving to be an effective strategy as this is the site of RSV infection (Davis et al., 2007). Recently, an anti-RSV nanobody ALX-0171 at 0.3 mg/kg dose was aerosolized using the AeroNeb Solo^®^ system; whereby 11% of the therapeutic nanobody was delivered to the lungs of RSV-infected lambs leading to reductions in both RSV lesions and viral antigen expression (Mora et al., 2018). In our study, the concentration of palivizumab delivered to the lungs as a single dose (~0.57–0.85 mg/ml) was 2–4 fold greater than that shown previously to neutralize RSV activity *in vitro* (Carlin et al., 1999). Aerosolized palivizumab also appeared to cause low-level inflammatory responses in the lung following delivery (data not shown) but interpretation of this finding is difficult as we did not have a control lamb without aerosolized palivizumab. Future studies are therefore necessary to fully document these effects.

Conventional delivery of palivizumab *via* intramuscular (IM) injection takes 3–5 days to achieve the dose in serum needed to protect against RSV infections (Sáez-Llorens et al., 1998). Rapid airway delivery of palivizumab may have substantial therapeutic benefits for high-risk infants and may provide more targeted control of RSV nosocomial infections in neonatal intensive care units (NICU) compared to IM palivizumab previously used for this purpose (Abadesso et al., 2004).

Despite the small sample size, this proof-of-concept study provides important data on the feasibility of pulmonary delivery and neutralizing bioactivity of palivizumab directly to the potential site of infection. This concept could also be tested on the new extended half-life RSV F mAb product (nirsevimab) that has recently been granted Breakthrough Therapy Designation by the US Food and Drug Administration (FDA). The lungs of a lamb constitute a highly translational model that shares many key features with that of a human infant, including structure and function. Determining the effect of inhaled palivizumab on RSV disease as well as the optimal inhaled dose/schedule necessary to prevent the potential emergence of RSV resistant strains are important next steps in the evaluation of this novel delivery strategy.

Demonstrating the efficacy of regional lung deposition of aerosolized palivizumab in a relevant model of human RSV infection would be valuable to explore its therapeutic potential. If successful, this strategy could be translated to high-burden settings where 99% of RSV-related deaths occur.

## Data Availability Statement

The datasets generated for this study are available on request to the corresponding authors.

## Ethics Statement

The animal study was reviewed and approved by the Murdoch Children’s Research Institute (MCRI) Animal Ethics Committee.

## Author Contributions

AR conceived the study design, obtained ethics approval, performed the experimental procedures, collected and processed the samples, and prepared the first draft of the manuscript. LD contributed to the design of the study, performed the experimental procedures, and critically revised the manuscript. MS, RBi, and DS assisted with sample collection. DS, MS, RBe, DV, RBi, WY, WH, and AM performed the experimental procedures and critically revised the manuscript. PE and LY contributed to and critically revised the manuscript. AR, LD, DS, RBe, DV, and PL were involved in the data analysis. PL and KM conceived the design of the study and critically revised the manuscript. All authors contributed to the article and approved the submitted version.

## Funding

This study is supported by a Jack Brockhoff Foundation Early Career Research Grant, an Australian Research Council Discovery Project Grant (DP170101061), a National Health & Medical Research Council (NHMRC) Development Grant (GNT1112870 and GNT1139340), a NHMRC Career Development Fellowship (GNT1165084), a National Health and Medical Research Council (NHMRC) Early Career Fellowship (GNT1123030), and the Victorian Government’s Operational Infrastructure Support Program.

## Conflict of Interest

LY and AM are listed as inventors on a patent related to the HYDRA device.

The remaining authors declare that the research was conducted in the absence of any commercial or financial relationships that could be construed as a potential conflict of interest.

## References

[B1] Abadesso C. Almeida H. II Virella D. Carreiro M. H. Machado M. C. (2004). Use of palivizumab to control an outbreak of syncytial respiratory virus in a neonatal intensive care unit. J. Hosp. Infect. 58, 38–41. 10.1016/j.jhin.2004.04.024 15350712

[B2] Carlin D. Pfarr D. S. Young J. F. Woods R. Koenig S. Johnson S. (1999). A Direct Comparison of the Activities of Two Humanized Respiratory Syncytial Virus Monoclonal Antibodies: MEDI-493 and RSHZl9. J. Infect. Dis. 180, 35–40. 10.1086/314846 10353858

[B3] Cheng Y. S. (2014). Mechanisms of pharmaceutical aerosol deposition in the respiratory tract. AAPS PharmSciTech 15, 630–640. 10.1208/s12249-014-0092-0 24563174 PMC4037474

[B4] Cortez-Jugo C. Qi A. Rajapaksa A. Friend J. R. Yeo L. Y. (2015). Pulmonary monoclonal antibody delivery via a portable microfluidic nebulization platform. Biomicrofluidics 9, 052603. 10.1063/1.4917181 25945147 PMC4393410

[B5] Davis I. C. Lazarowski E. R. Chen F.-P. Hickman-Davis J. M. Sullender W. M. Matalon S. (2007). Post-Infection A77-1726 Blocks Pathophysiologic Sequelae of Respiratory Syncytial Virus Infection. Am. J. Respiratory Cell Mol. Biol. 37, 379–386. 10.1165/rcmb.2007-0142OC PMC2084468 17541010

[B6] Do L. A. H. Tse R. Nathanielsz J. Anderson J. Ong D. S. Chappell K. (2019). An Improved and High Throughput Respiratory Syncytial Virus (RSV) Micro-neutralization Assay. J. Visualized Experiments 143. 10.3791/59025 30741261

[B7] Finlay W. H. (2019). “Particle size distributions,” in The Mechanics of Inhaled Pharmaceutical Aerosols 2nd edition. (London: Elsevier).

[B8] Kilkenny C. Browne W. J. Cuthill I. C. Emerson M. Altman D. G. (2010). Improving bioscience research reporting: the ARRIVE guidelines for reporting animal research. PloS Biol. 8, e1000412–e1000412. 10.1371/journal.pbio.1000412 20613859 PMC2893951

[B9] Mora A. Detalle L. Gallup J. M. Van Geelen A. Stohr T. Duprez L. (2018). Delivery of ALX-0171 by inhalation greatly reduces respiratory syncytial virus disease in newborn lambs. mAbs 10, 778–795. 10.1080/19420862.2018.1470727 29733750 PMC6150622

[B10] Nair H. Nokes D. J. Gessner B. D. Dherani M. Madhi S. A. Singleton R. J. (2010). Global burden of acute lower respiratory infections due to respiratory syncytial virus in young children: a systematic review and meta-analysis. Lancet 375, 1545–1555. 10.1016/S0140-6736(10)60206-1 20399493 PMC2864404

[B11] O’Brien K. L. Baggett H. C. Brooks W. A. Feikin D. R. Hammitt L. L. Higdon M. M. (2019). Causes of severe pneumonia requiring hospital admission in children without HIV infection from Africa and Asia: the PERCH multi-country case-control study. Lancet 394, 757–779. 10.1016/S0140-6736(19)30721-4 31257127 PMC6727070

[B12] Rajapaksa A. E. Ho J. J. Qi A. Bischof R. Nguyen T.-H. Tate M. (2014). Effective pulmonary delivery of an aerosolized plasmid DNA vaccine via surface acoustic wave nebulization. Respiratory Res. 15, 1–12. 10.1186/1465-9921-15-60 PMC4040411 24884387

[B13] Rezk A. R. Tan J. K. Yeo L. Y. (2016). HYbriD Resonant Acoustics (HYDRA). Adv. Mater. 28, 1970–1975. 10.1002/adma.201504861 26743122

[B14] Sáez-Llorens X. Castaño E. Null D. Steichen J. Sánchez P. J. Ramilo O. (1998). Safety and pharmacokinetics of an intramuscular humanized monoclonal antibody to respiratory syncytial virus in premature infants and infants with bronchopulmonary dysplasia. Pediatr. Infect. Dis. J. 17, 787–791. 10.1097/00006454-199809000-00007 9779762

